# Molecular and Physiological Adaptations to Seasonal Training in Elite U18 Ice Hockey Players

**DOI:** 10.3390/sports14020057

**Published:** 2026-02-04

**Authors:** Attila Czont, Zsolt Bodor, Tamás Koncsag, Ildikó Miklóssy

**Affiliations:** 1 Faculty of Science, University of Pecs, Ifjusag 6, H-7624 Pecs, Hungary; czontattila@uni.sapientia.ro (A.C.); koncsagtamas@uni.sapientia.ro (T.K.); 2 Department of Bioengineering, Sapientia Hungarian University of Transylvania, Piata Libertatii, 530104 Miercurea Ciuc, Romania; bodorzsolt@uni.sapientia.ro

**Keywords:** U18 ice hockey, irisin, cfDNA

## Abstract

Monitoring adolescent team-sport athletes may benefit from combining performance and molecular markers, but empirical evidence supporting this approach in youth team sports remains limited. Objective: Our study investigated molecular and physiological adaptations to seasonal training in elite U18 ice hockey players, focusing on aerobic capacity, salivary cortisol, serum irisin, and cell-free DNA (cfDNA) dynamics. Methods: National-level U18 players were enrolled in our study (*n* = 23 for cross-sectional analysis, *n* = 12 longitudinal) during the pre- and early-competition season. Aerobic performance was assessed via graded treadmill VO_2_max testing, and the biochemical markers quantified using ELISA-based assays. Results: From pre- to early-season (paired *n* = 12), VO_2_max increased by 10.6% (g = +1.00, *p* = 0.003) and irisin by 14.7% (g = +0.83, *p* = 0.010). cfDNA decreased by 60.8% (g = −0.54, *p* = 0.070; moderate effect, not statistically clear), while cortisol remained stable (+11.3%; *p* = 0.667). Inter-individual variability increased for VO_2_max and irisin and decreased by 82% for cfDNA. Exploratory cross-sectional positional analysis indicated higher irisin levels in forwards and elevated cfDNA in defensemen, although differences did not reach statistical significance. Conclusions: These preliminary findings provide cohort-size limited longitudinal evidence of chronic irisin elevation in ice hockey players and highlight the possibility of combining VO_2_max + irisin + cfDNA to assist individualized load/recovery in elite youth ice hockey.

## 1. Introduction

Ice hockey is a high-intensity intermittent team sport requiring both aerobic and anaerobic fitness to sustain repeated maximal efforts, rapid accelerations, and high-force physical contacts. As seasonal training induces quantifiable body composition and metabolic adaptations in ice hockey players, the high demands in elite U18 ice hockey require monitoring to evaluate both performance capacity and physiological stress. While external load metrics and field tests are widely implemented, fewer studies integrate molecular with physiological indicators to characterize seasonal adaptation in adolescent team sports. Regarding performance in ice hockey, as in other sports, VO_2_max is a central marker of aerobic capacity, with professional players typically achieving values around 55.9 ± 5.2 mL·kg^−1^·min^−1^ [1], with elite youth hockey players showing an increase in absolute VO_2_max with age and physical maturation [2]. Higher VO_2_max is associated with reduced fatigue during repeated skating sprints and faster recovery between shifts [3], yet above a minimal threshold it does not predict season or career statistics in elite adult athletes [1]. Earlier studies showed that high-intensity interval training interventions consistently improved VO_2_max [2].

Reports on seasonal adaptations in ice hockey are heterogeneous. Several works describe maintenance or decline in aerobic fitness across the competitive calendar, underscoring the need for ongoing maintenance work during the season [4,5]. Pre-/off-season programs are typically associated with lower body fat and greater lean mass that can persist into competition [5,6]. Post-exercise blood lactate often decreases from pre- to mid-season and it may rise again by the season end, potentially reflecting accumulated fatigue [5]. Reported longitudinal changes are somewhat inconsistent across studies [7], that reflect the importance of periodized in-season conditioning and monitoring, including molecular biomarkers, to manage load, preserve fitness, and minimize overtraining. Accordingly, we focused on two complementary biomarkers, understudied longitudinally in a sport-specific context like adolescent team sports.

The recently characterized exerkine irisin released from skeletal muscle has been linked to adipose tissue browning and improved metabolic regulation [8]. The molecular pathways of irisin production through the PGC-1α/FNDC5 pathway are well-established: exercise induces the expression of peroxisome proliferator-activated receptor gamma co-activator 1-α (PGC-1α) in skeletal muscle, which promotes the cleavage of fibronectin type III domain-containing protein 5 (FNDC5) to irisin [9]. Irisin functions as an exercise-induced myokine that mediates communication between muscles and other tissues [10,11], while its secretion is strongly regulated by exercise intensity and duration [9,10,12,13]. However, most studies have analyzed only acute effects. Literature suggests that there is an interplay between irisin levels, metabolic adaptations to exercise, body composition, and overtraining syndrome [11,14]. Thus, irisin seems a promising marker for understanding the physiological adaptations to exercise, but its role in adolescent sports-specific athletic performance remains mainly unexploited.

Exercise also induces sharp acute increases in cell-free DNA (cfDNA), another interesting, but under-explored molecular marker for exercise science. The predominant source is granulocytes, implicating immune activation and oxidative stress [15,16]. Some studies found sustained cfDNA elevations following repeated or prolonged high-intensity exercise in relation to chronic strain and potential overtraining [17,18]. cfDNA correlates strongly with markers such as creatine kinase and myoglobin [18,19] and demonstrates faster kinetics than traditional muscle damage markers, making it a sensitive indicator of acute load [20].

Competitive ice hockey also elicits robust activation of the hypothalamic–pituitary–adrenal (HPA) axis. Acute increases may reflect both physical and psychological stress. Studies show various chronic effects depending on the training regimen or observation period [21,22,23]; thus, monitoring can provide insights into an athlete’s overall well-being and training status [24,25].

Evidence regarding these biomarkers in youth team sports is limited and often acute or cross-sectional, leaving it uncertain how these biomarker candidates behave from pre-season to competition and how they relate to aerobic adaptation and endocrine stress.

Thus, during our study, we profiled VO_2_max, serum irisin, serum cfDNA, and salivary cortisol from pre-season to early competition in elite level U18 ice hockey players to capture changes in fitness, myokine signaling, cellular stress, and HPA activity under an applied program. Based on literature data, the high intensity intermittent nature of the sport, and the OPT model based complex resistance training protocol, we hypothesized increases in VO_2_max and irisin, a decrease in cfDNA, and relatively stable cortisol under the given training regimen. Exploratory analyses assessed positional differences and inter-individual variability to inform biomarker-guided evaluation in high-intensity intermittent sports.

## 2. Materials and Methods

### 2.1. Study Design

This study involved a national-level U18 ice hockey team (Szeklerland Ice Hockey Academy, SZJA, Romania) investigated during the 2023/2024 pre-season (June) and early-season (September). A schematic summary of our study, showing assessment design and experimental framework is presented in the Supplementary File (Figure S1).

Our study group consisted of 23 athletes (14 forwards, 5 defensemen, and 4 goalies), aged 15.6 ± 0.561, weight 69.980 ± 9.62 kg, height 176.746 ± 5.271 cm. Twenty-six athletes were initially enrolled; three were excluded (3 assay QC), yielding *n* = 23 for cross-sectional summaries and *n* = 12 for paired longitudinal analyses. All players were enrolled in a residential academy with a standardized, supervised diet, which was expected to reduce diet-related intra-group variability in the biochemical markers.

Data collection was conducted at the SZJAs Medical and Methodological Center. Informed consent from all participants was collected and the research was approved by the Bioethics Committee of the Sapientia Hungarian University of Transylvania, nr. 1/03.01.2023.

### 2.2. Training Regimen

Pre-season training was a five-phase regimen based on the OPT model developed by NASM; each session was composed of a main part, as well as segments dedicated to warm-up, core strength and balance, plyometrics, speed and agility, and a cool-down, each with varying intensity. The training structure included stability for 1.5 weeks, strength-endurance for 2 weeks, hypertrophy for 2.5 weeks, maximum strength for 3 weeks, and explosiveness for 3 weeks, with rest intervals between phases (weekly 4 × 2 h). Additionally, running and cycling endurance sessions (weekly 3 × 50–70 min) targeted different energy systems: aerobic (4 weeks), anaerobic alactacid (4 weeks), and anaerobic lactacid (4 weeks), and athletes had weekly 4 × 2 h on-ice technical and tactical training.

### 2.3. Anthropometry

Body height was determined in barefoot height (±0.1 cm) using a wall mounted stadiometer. All measurements were performed by authorized personnel. Body weight was measured in the morning, two hours after a light breakfast. Participants did not train or take medications prior to the measurements. Defensemen were aged 15.5 ± 0.548, weight 72.1 ± 12.306 kg, height 175.23 ± 4.657 cm. Forwards were aged 15.625 ± 0.5, weight 67.44 ± 7.207 kg, height 176.70 ± 5.243 cm.

### 2.4. Aerobic Performance

Aerobic performance was assessed using the Quark CPET (Cosmed, Rome, Italy) system by a standard incremental maximal oxygen uptake test, performed until voluntary exhaustion on a treadmill ergometer platform. A standard Bruce protocol was performed [3], exhaled air was continuously sampled by the gas analyzer (CPET Cosmed, Rome, Italy), and the rate of oxygen uptake (VO_2_), carbon dioxide production (VCO_2_), minute ventilation (VE), and the respiratory exchange ratio (RER) were recorded breath by breath, by an on-line software (Omnia Cosmed, Rome, Italy). The VO_2_max test was stopped by the exercise performer himself due to subjective fatigue (influenced by factors like motivation, perseverance), validation criteria for the maxima test: RER greater than 1.10 at test termination, oxygen uptake reaching a plateau or starting to fall even though the work rate kept increasing, and achieving maximal age-specific heart rate.

### 2.5. Biochemical Markers

Saliva and blood samples were collected from all athletes in the morning between 7.00 and 9.00, after a 24 h rest period without structured training, at both pre-season and early-season time points, to minimize circadian variation in cortisol and circulating biomarkers.

Saliva samples were collected 30 min after consuming any food, preceded by water rinse and by passive drooling in sterile centrifuge tubes. Samples were kept at −80 °C until further analysis. Salivary cortisol concentration was determined by ELISA (LDN, Nordhorn, Germany, cat. nr. SA-E-6000), according to the manufacturer’s instructions.

Blood samples were collected at the same timepoints in EDTA tubes, and serum fraction was obtained by incubating the samples for 2 h at 22 °C, followed by centrifugation for 15 min at 1000× *g*. Supernatants were collected and aliquoted for storage at −80 °C until assay. Serum irisin and cell-free DNA levels were determined using Human Irisin ELISA Kit (Finetest, Wuhan, China, cat. nr. EH4702) and the Human Anti-Cell Free DNA Kit (Finetest, Wuhan, China, cat. nr. EH4983), according to the manufacturer’s instructions. Plates included 6-point standard curves and controls. LOD/LOQ: irisin 0.938 ng/mL, 1.563 ng/mL; cfDNA 0.938 ng/mL, 1.563 ng/mL; cortisol 0.019 ng/mL, 0.1 ng/mL. Out-of-range samples were diluted and re-run. Detection was carried out on a Ledetect96 microplate reader (Labexim Products, Lengau, Austria). Data were fitted on a 4-parameter logistic curve model.

### 2.6. Statistical Analysis

All statistical analyses were performed using R (R4.2.1), unless otherwise specified. Descriptive statistics and data visualization were conducted in R. Data normality was assessed using the Shapiro–Wilk test performed at the variable level (Table S1). Spearman’s rank and Pearson’s correlation coefficients were calculated to determine the relationships between variables, depending on the data distribution characteristics. Descriptive statistics included the minimum, 25th percentile (Q1), median, mean, 75th percentile (Q3), and maximum values.

Depending on the distribution and the type of data, statistical significance was evaluated using one-way ANOVA followed by Tukey’s Honest Significant Difference (TukeyHSD) post hoc test, Kruskal–Wallis test, or paired *t*-tests, as appropriate. Effect sizes were expressed as Cohen’s d or Hedges’ g values. All statistical analyses were conducted using a significance level of *p* < 0.05.

## 3. Results

Our results provide a comprehensive analysis of the physiological adaptations, performance metrics, and biochemical responses observed in elite U18 ice hockey players during the pre-season and early-season phase.

### 3.1. Cross-Sectional Analysis of Physiological and Biochemical Parameters

**Figure 1:**
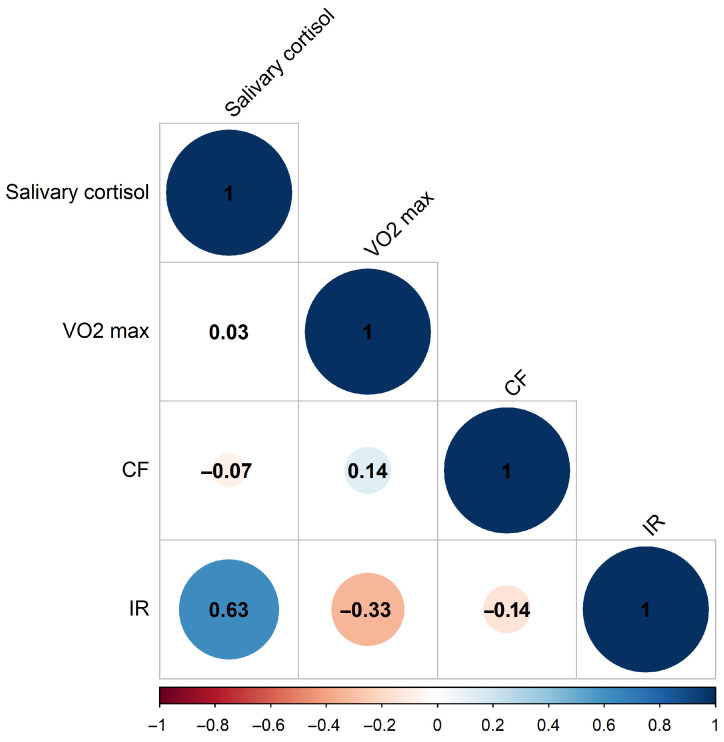
Pearson correlation coefficients of measured biochemical and physiological parameters at pre-season start. (Blue: positive correlation, Red: negative correlation. CF: cell-free DNA, IR: irisin, *n* = 23).

We started by assessing the players’ anthropometric measurements, aerobic performance, and biochemical markers to establish baseline data for subsequent analysis. Biological samples were obtained at rest, after 24 h without structured training, and before exercise testing; thus, the observed changes reflect baseline/resting values rather than acute post-exercise spikes. Our study group consisted of 23 athletes (14 forwards, 5 defensemen, and 4 goalies), aged 15.65 ± 0.561 years, weight 69.980 ± 9.62 kg, height 176.746 ± 5.271 cm. In the first set of measurements, a pre-season cross-sectional analysis was performed on the team’s basic biochemical and aerobic performance data. Next, we performed as a strictly exploratory analysis, due to the limited sample and unbalanced subgroup size, on the correlations and differences between biomarker patterns based on playing position. Given that the data are normally distributed (Table S1), we used the Pearson correlation for evaluation of cross-sectional data. Pearson correlation coefficients suggest (Figure 1) significant positive correlation (0.63) between serum irisin and salivary cortisol data, while VO_2_max and serum irisin tend towards negative correlation (−0.33) on this population scale (*n* = 23).

**Figure 2:**
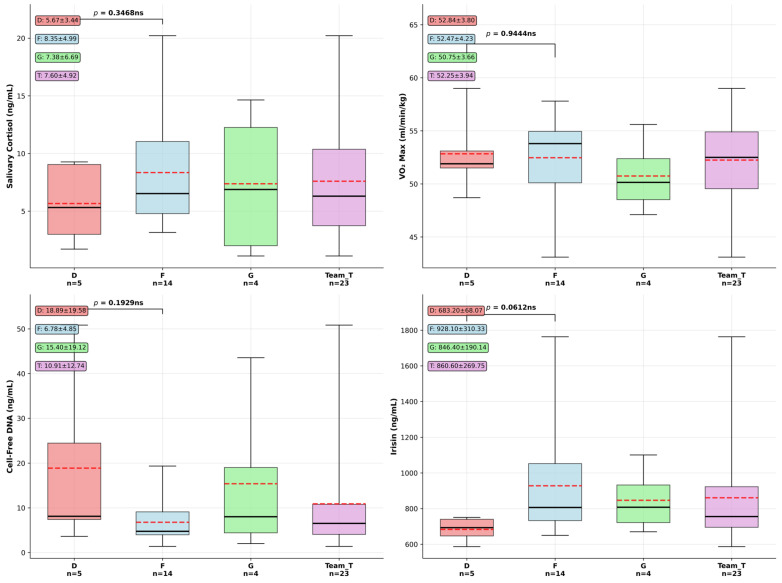
Exploratory results of biomarker comparison between hockey playing positions. Box plots show median, interquartile range, and individual outliers for defensemen (D, *n* = 5), forwards (F, *n* = 14), goalies (G, *n* = 4), and total team (T, *n* = 23). Dashed lines represent group means. Data are presented as median (solid lines), interquartile range (box), and whiskers extending to 1.5 × IQR. One-way ANOVA revealed no significant differences between D and F positions (all *p* > 0.05). Data presented as mean ± SD in text boxes.

Playing position, especially in such a highly demanding team sport could be reflected in physiological performance markers, so our next question was if differences appear between our F (forwards) and D (defensemen) groups. We conducted an exploratory analysis on data available for the two groups (see descriptive statistics and one way ANOVA significance testing results in Supplementary Table S2), and used box plots to visually summarize the distribution, central tendency, and variability of the data (Figure 2). Specifically, they illustrate the median (Q2), interquartile range (Q1–Q3), and the full data range (minimum to maximum). This visualization facilitates the identification of outliers, skewness, and differences between groups, thereby supporting the interpretation of statistical trends and patterns within the dataset. Distribution and differences between defensemen (D) and forwards (F) were studied first. Effect sizes expressed in Cohen’s d (Supplementary Table S2) were higher in the case of cfDNA of −1.51 and 1.2 in the case of irisin (mean values being D: 683.2 ± 68.07 ng/mL and F: 928.1 ± 310.33 ng/mL, *p* = 0.0612). Forwards registered both higher levels and greater individual variability in their exerkine response compared to defensemen. Non-significant but notable patterns between groups also emerged during our analysis considering serum cell-free DNA difference (*p* = 0.1929, D: 18.8 ± 15.7 ng/mL, F: 6.78 ± 4.85 ng/mL). There were no significant differences identified between D and F groups related to salivary cortisol (*p* = 0.346) levels, where forwards showed somewhat higher stress hormone levels. Similarly, we observed no meaningful difference in cardiovascular fitness between positions, (D: 52.84 ± 3.8 mL·kg^−1^·min^−1^, F: 52.47 ± 4.2 mL·kg^−1^·min^−1^, Cohen’s d = −0.15, *p* = 0.944).

Based on the exploratory biomarker analysis comparing defensemen (*n* = 5) and forwards (*n* = 14) in elite hockey players, effect size calculations revealed varying magnitudes of difference between positional groups despite the lack of statistical significance. Salivary cortisol levels demonstrated a medium effect size (Cohen’s d = 0.73), with forwards showing higher stress hormone concentrations (8.35 ± 4.99 ng/mL) compared to defensemen (5.67 ± 3.44 ng/mL), suggesting potentially meaningful physiological differences in stress response patterns. Cell-free DNA concentrations exhibited the largest effect size (Cohen’s d = −1.51), representing a large magnitude difference with defensemen, displaying substantially higher cellular stress markers (18.89 ± 19.58 ng/mL) versus forwards (6.78 ± 4.85 ng/mL), potentially reflecting greater physical trauma exposure in defensive play. Irisin levels showed a large positive effect size (Cohen’s d = 1.2), with forwards demonstrating markedly elevated concentrations (928.10 ± 310.33 ng/mL) compared to defensemen (683.20 ± 68.07 ng/mL), suggesting enhanced metabolic adaptation responses in forward players. These effect size analyses reveal that while statistical significance was not achieved due to limited sample sizes, meaningful physiological differences could exist between playing positions, particularly in cellular stress markers and exercise-induced myokine responses. In conclusion, key highlights of this cross-sectional team and position-based analysis reveal only weak correlations between the studied parameters on the whole team level, while non-significant position-specific differences, expressed as effect sizes, seem to be present, as forwards had higher exercise-induced hormone response (irisin), while defensemen showed higher cfDNA values. Cardiovascular fitness is equivalent between positions, and stress responses represented by single salivary cortisol levels vary but not significantly between positions.

### 3.2. Longitudinal Biomarker Analysis

**Figure 3:**
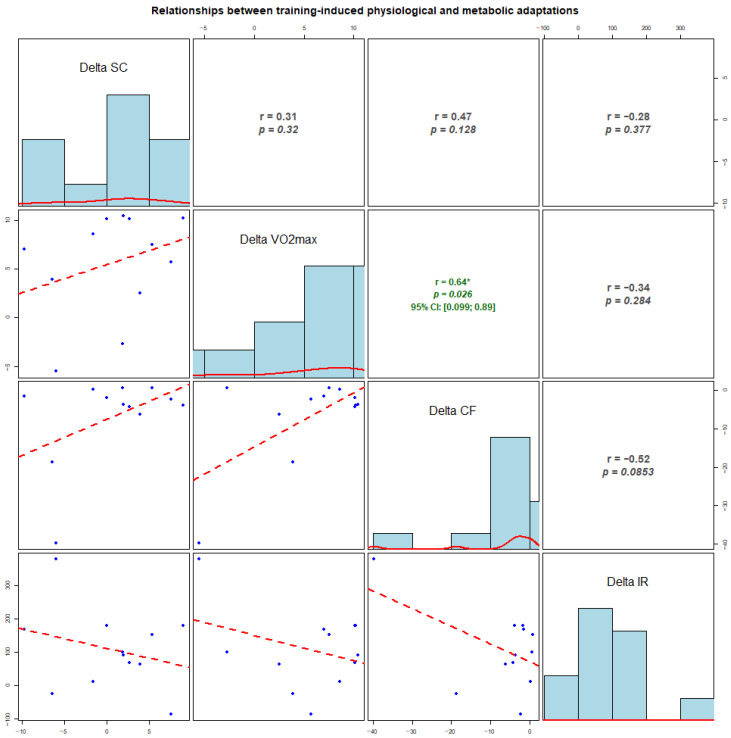
Correlations between individual changes (delta) in physiological and molecular markers from pre-season to early-season (*n* = 12). Delta values represent early-season minus pre-season (early − pre) for salivary cortisol, VO_2_max, cell-free DNA (cfDNA), and irisin. The diagonal panels show histograms of the distribution of Δ for each variable. The lower panels display scatterplots (blue dots) with fitted regression lines (red dashed lines). The upper panels present Pearson correlation coefficients (r) and corresponding *p*-values. Where applicable, 95% confidence intervals (CI) are shown. Asterisks indicate statistically significant correlations (*p* < 0.05).

In our second set of experiments, we tried to identify correlation strengths and directions between the measured biochemical and physiological parameters; namely, we performed exploratory analyses of correlations between individual changes (Δ, early-season–pre-season) in VO_2_max and biomarkers (Figure 3). No clear associations were observed for changes in cortisol with any variable (all *p* > 0.12). Changes in VO_2_max showed a moderate positive correlation with changes in cfDNA (r = 0.64, *p* = 0.026), while a non-significant, moderate-sized inverse association between ΔcfDNA and Δirisin was observed (r = −0.52, *p* = 0.085). Given the small sample (*n* = 12), the analyses are exploratory and illustrate the relationships between individual adaptation profiles across markers, while the correlations should be interpreted cautiously.

**Figure 4:**
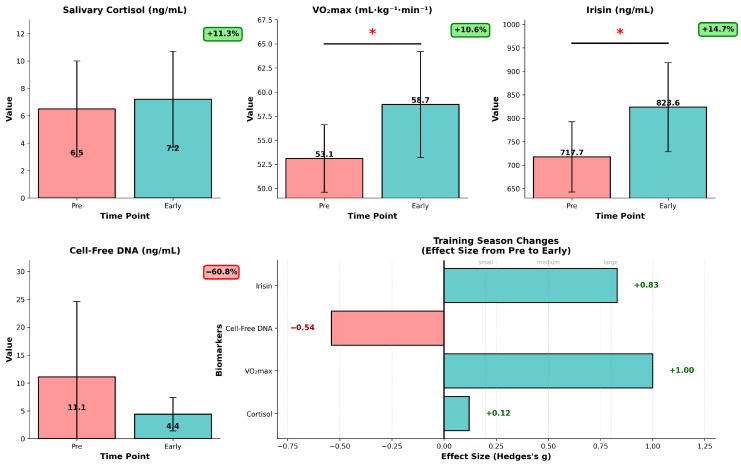
Longitudinal biomarker analysis for training season progression in U18 ice hockey players (*n* = 12). Data are presented as mean ± SD, red bars represent pre-season values, teal bars represent early-season values, individual values are overlaid. * *p* < 0.05 (paired *t*-test, pre- vs. early-season). Salivary cortisol (ng/mL) showing non-significant increase (+11.3%, *p* = 0.667), VO_2_max (mL·kg^−1^·min^−1^) demonstrating significant improvement (+10.6%, *p* = 0.003), irisin levels (ng/mL) with significant elevation (+14.7%, *p* = 0.010), cell-free DNA (ng/mL) showing reduced baseline, moderate effect (−60.8%, *p* = 0.070). The low right panel shows corresponding Hedges’ g effect sizes (Δ = early − pre), with horizontal reference lines for small (0.2), medium (0.5), and large (0.8) effects.

A comprehensive longitudinal biomarker analysis revealed significant training adaptations from pre-season to early-season. Key insights from our analysis show statistically significant changes (*p* < 0.05): first, in the case of VO_2_max—+10.6% improvement (*p* = 0.003), pre-season—53.06 ± 3.28 mL·kg^−1^·min^−1^, early-season—58.71 ± 5.03 mL·kg^−1^·min^−1^. This shows, as presumed by coaching staff, substantial cardiovascular fitness improvement (Figure 4). To get a better insight into this important fitness marker, we analyzed dVO_2_max data as VO_2_max response, which can offer a deeper understanding in training efficiency than absolute values. A box plot of dVO_2_max values was generated to assess the distribution of the observed differences (Figure S2), and it reveals that most players show positive changes in VO_2_max (5.56 mL·kg^−1^·min^−1^ mean improvement), the distribution indicating a stable general fitness improvement from pre-season to early-season.

In the case of irisin, a myokine which, to our knowledge, has not been studied in ice hockey players, we saw an increase of 14.7% (*p* = 0.01), our mean data ranging from pre-season mean of 718.7 ± 65.2 ng/mL to 824.6 ± 103.2 ng/mL values early-season. This chronic effect suggests enhanced exercise-induced myokine response in our experimental setting (Figure 4). We observed a non-significant tendency in the case of cell-free DNA, used as a cellular damage marker, recording a −60.8% reduction (*p* = 0.07) in serum concentrations; pre-season mean values of 11.10 ± 13.82 ng/mL decreased to 4.35 ± 2.52 ng/mL (95%CI −0.6–14.13, Table S3), which might suggest potential reduction in cellular damage/stress due to physiological adaptation to the training regimen. The salivary cortisol levels seem to suggest the team maintained stable stress hormone levels despite the training intensity. The salivary cortisol levels, however, might show individual and diurnal changes which could not be detected in our experimental setting. Regarding variability in responses, inter-individual variability of VO_2_max (+53% in SD) and irisin (+58% in SD) responses increased, and in contrast, variability in cell-free DNA concentrations was reduced by 82%, while cortisol variability mainly remained unchanged (−9% in SD).

Overall, our data suggest that the pre-season conditioning program (linear periodized OPT-based concurrent strength and conditioning with aerobic/alactic/lactic energy system development) was effective; athletes exhibited positive adaptations to the imposed training stress, as evidenced by improvements in key aerobic performance and physiological markers. These patterns are consistent with enhanced recovery and reduced cellular stress, as reflected by lower resting cfDNA.

## 4. Discussion

From the pre-season to the early competition stage, the studied elite U18 ice-hockey players demonstrated a significant increase in VO_2_max and irisin concentration and a reduction in cfDNA, while salivary cortisol remained stable. These changes might suggest improved cardiorespiratory fitness, a persistent exercise-induced myokine signal, and lower resting cellular stress without evidence of chronic HPA-axis activation. Exploratory positional patterns (higher irisin in forwards; higher cfDNA in defensemen) were plausible but underpowered; larger samples with position-specific exposure metrics are needed to confirm this.

### 4.1. Seasonal Adaptations in VO_2_max, Irisin, Cell-Free DNA, and Cortisol

During a typical game, ice hockey players perform 4–8 maximal sprints per shift, accumulating approximately 4–8 min of skating at 70–90% of maximal heart rate and 18–22 min of mixed aerobic and anaerobic exertion [1,26]. Energy contribution during sprint phases is dominated by anaerobic glycolysis (~69%), whereas recovery intervals are predominantly aerobic (~31%) [3]). Thus, pre-season training involves a combined training regimen to prepare athletes for both aerobic and anaerobic performance. Our early-season sampling likely captures the initial positive adaptation window observed after intensive pre-season periods. The longitudinal data represent the primary contribution of this study. VO_2_max increased by 10.6% (53.1 → 58.7 mL·kg^−1^·min^−1^, *p* = 0.003, g = +1.00). This aligns with reports of early-season improvements in aerobic capacity and metabolic efficiency yet contrasts with studies showing maintenance [27] or decline by late season—patterns often attributed to residual fatigue, and constrained recovery [7]. Seasonal changes in collegiate ice hockey players were studied by [5], where aerobic fitness measured as heart rate during the minutes 3–4 of the submaximal test increased from pre- to end- and from mid- to end-season (*p* ≤ 0.05), while post-exercise blood lactate fell from pre- to mid-season (9.3 → 6.2 mmol·L^−1^) and rose from mid- to end-season (6.2 → 8.0; *p* ≤ 0.05). Similar patterns were observed in high level age-group ice hockey players (13–14 years), where maximal aerobic power expressed in mL·kg^−1^·min^−1^ was maintained throughout the hockey season, but improved during off-season [28]. It is also noteworthy to mention that high-intensity interval training interventions have consistently improved VO_2_max in ice hockey players [29,30,31]. These mixed findings underline the importance of specific pre-season and in-season conditioning and ongoing monitoring to preserve fitness across the competitive calendar.

More importantly, our study provides novel longitudinal evidence of molecular adaptations in elite U18 ice hockey players during pre-seasonal training. The observed chronic 14.7% increase in irisin (718.7 → 824.6 ng/mL, *p* = 0.010, g = +0.83) represents, to the best of our knowledge, the first such report in ice hockey athletes. Acute exercise-induced irisin elevations are well established in the literature [12]. Earlier studies reporting lower baseline irisin levels in older vs. younger and sedentary vs. active, with acute exercise-induced increase being unaffected by age or fitness but positively correlated with swimming intensity in adolescents [32]. It was, similarly, not affected by training status after acute endurance exercise, while running was found to sustain higher and more sustained irisin levels than cycling [33]. Interestingly, exercise in a cold environment (ice swimming) produced a decrease in irisin acute response [34]. Adjusted for insulin resistance, elite athletes were shown to have higher irisin levels than sedentary subjects, and irisin correlated with more metabolic markers as training status increased [35]. In rowing, with athletes of a similar age to our group, plasma irisin concentrations were increased (by 8%; *p* = 0.013) immediately after aerobic rowing exercise [36]. In team sports, high acute irisin response was shown after football matches [37] and after handball training in adolescent elite level players [38]. However, some studies indicated acute high-intensity swimming does not induce irisin response in adolescent athletes [39], and that neither acute nor short-term chronic high-intensity resistance training impacts circulating irisin levels in healthy young adults [40]. On the other hand, resting irisin concentrations were found higher in combat athletes than in sedentary young males and similarly in athletes performing aerobic endurance exercise (footballers) or dynamic sports compared to strength training or static sports (bodybuilders) [41].

Chronic irisin adaptations remain mainly under-studied in athletic populations and debated because of substantial variations by training modality, population, and measurement methods, especially for ELISA. In non-athlete healthy populations, aerobic and resistance training can elevate irisin levels, with high-intensity resistance training (HIRT) producing a more pronounced increase than traditional endurance exercise [42,43]. HIIT using the Tabata protocol increased irisin by 29.7% in trained men [44], others reporting no chronic effect but higher resting irisin in younger groups [45]. A meta-analysis by [46] confirmed that exercise interventions, by subgroup analyses, showed significant increases following resistance and combined aerobic + resistance protocols, aerobic training alone producing no significant change. Contrary to our findings, a study employing a similar cohort of 15 3rd league football players (age ± 22) found no significant difference in irisin levels after 3 months from the start of the competitive season [47]. The same conclusion was drawn in the case of elite male rowers, where irisin levels were not sensitive to the elevated training stress after a 6-month volume-extended training period [48]. Moreover, one study [49] found that seasonal training variations did not significantly alter most resting physiological and biochemical parameters, including irisin, in elite cyclists during a 6-month macrocycle. Irisin showed some isolated correlations: a higher tendency of its concentration was revealed in the pre-competition and competition period compared to the baseline stage, and a significant positive correlation with cortisol was found in the first phase of the training (r = 0.758, *p* = 0.018).

Systematic reviews reveal substantial heterogeneity among studies. A meta-analysis of resistance training trials (*n* = 282 pooled) found a non-significant trend toward increased irisin, and a significant increase in demanding and progressive training programs (*p* = 0.03 [50], while another meta-analysis showed exercise increased irisin overall (SMD = 0.957), with HIIT producing the largest effect in obese populations [51]. These contradictory findings likely reflect methodological differences (assay types, training protocols, baseline fitness), population characteristics (age, obesity status), and exercise intensity [12,51,52]. Given ongoing uncertainty regarding irisin ELISA specificity and inter-assay variability, our primary focus is on within-study longitudinal changes.

In our study framework, the applied training regimen plausibly augmented PGC-1α/FNDC5 signaling, yielding a seasonal increase in circulating irisin at rest. Given the role of irisin in inter-organ communication and metabolic remodeling, a sustained rise across weeks—not only acute bouts—may be consistent with a shift toward a more oxidative, fatigue-resistant phenotype, although we did not directly assess muscle phenotype or substrate utilization. Our sustained seasonal rise in young, highly trainable ice hockey players aligns with results in HIIT and HIRT-based interventions in healthy populations and may reflect the high-intensity intermittent nature of ice hockey training.

Resting cell-free DNA showed a moderate effect size, but statistically a non-significant reduction of 60.8% (11.1 → 4.4 ng/mL, *p* = 0.070, g = −0.54) in the studied group, which might suggest reduced systemic stress and improved physiological adaptation. This finding is particularly novel, as most cfDNA research focuses on acute exercise-induced spikes rather than chronic baseline adaptations. Recent evidence distinguishes these responses: acute exercise triggers primarily neutrophil-derived cfDNA release, while chronic training can alter resting cfDNA levels. An 8-month endurance intervention (moderate intensity endurance training) in adults with cardiovascular risk factors (*n* = 98) decreased resting cfDNA in those who improved fitness, alongside increased DNase activity [53]. Conversely, acute maximal [54] and chronic high-load or overtraining can increase resting cfDNA. Male boxers (*n* = 13) showed ~4-fold elevations in baseline cfDNA after high-load training blocks, associated with oxidative stress and muscle damage [55]. According to [33], acute irisin release was also negatively, though not significantly, related to muscle-damage markers (aspartate aminotransferase and myoglobin), consistent with our results. This might suggest that an objectively periodized, well-tolerated training may reduce cfDNA, while excessive load can elevate it. Our 60.8% reduction may be consistent with a repeated-bout effect [56] and effective training periodization, while the 82% decrease in inter-individual variability may indicate standardized recovery across athletes. However, the marginal statistical significance (*p* = 0.070), the wide confidence interval, as well as the limited chronic cfDNA literature indicate cautious interpretation and replication in larger cohorts.

Salivary cortisol remained stable (6.5 → 7.2 ng/mL, *p* = 0.667, g = −0.12), differing from studies reporting chronic elevations in overtrained athletes [57]. This stability, combined with improved aerobic performance and reduced cfDNA is compatible with effective load management and appropriate training periodization. However, cortisol is highly sensitive to diurnal rhythm and sampling conditions, thus future studies should incorporate multiple time points to better capture HPA-axis dynamics.

### 4.2. Exploratory Results on Ice Hockey-Specific Positional Demands

Ice-hockey specific positional roles impose distinct physiological demands. Forwards sustain greater volumes of high intensity skating and present higher post-shift blood lactate concentrations than defensemen [58]. High-intensity skating relies heavily on glycogen, leading to substantial depletion even during short shifts; thus forwards, who engage in more high-intensity skating, may benefit more from higher aerobic capacity compared to defensemen [59]. Defensemen typically engage in more physical contact, body checking, and defensive battles along the boards, activities associated with greater mechanical stress and potential cellular damage.

In our exploratory cross-sectional analysis (*n* = 23), forwards showed non-significant elevation in irisin concentrations (928.1 ± 310.3 vs. 683.2 ± 68.1 ng/mL, *p* = 0.0612), which might suggest consistency with evidence of intensity-dependent irisin secretion [10,32]. No positional differences were observed in VO_2_max or cortisol, contrary to a three decade-long summarizing study of collegiate athletes [4], where forwards had a higher relative peak oxygen consumption compared with defensemen (58.7 ± 4.7 vs. 57.2 ± 4.4 mL·kg^−1^·min^−1^) (*p* = 0.032). The elevated cfDNA in defensemen may reflect greater physical contact and sustained defensive efforts. Our observations are merely hypothesis-generating, require confirmation in larger cohorts, while position-balanced samples with exposure metrics are needed to confirm these patterns.

### 4.3. Limitations and Practical Applications

Our study is limited by a modest longitudinal sample size (*n* = 12), and small, unbalanced positional subgroups (forwards *n* = 14, defensemen *n* = 5), which considerably reduces the statistical power and increases the risk of both Type I and Type II errors. Consequently, position-specific differences and correlation patterns must be interpreted as exploratory and hypothesis-generating. Given the small sample size and exploratory intent, we did not apply formal corrections for multiple comparisons. Accordingly, *p*-values—particularly for correlation analyses—should be interpreted descriptively.

Absence of late-season measurements and lack of direct training load quantification (e.g., heart-rate-derived internal load, session-RPE, GPS-derived external load or detailed volume/intensity logs) are another limitation of our study. Future research should incorporate serial assessments across the entire competitive season, examine dose–response relationships between training load with established internal and external training load measures, biomarker and body composition changes, and explore associations with performance outcomes and injury incidence.

Assay-related variability and uncertainties, especially regarding irisin ELISA specificity further limit cross-study comparison of absolute values [52]; therefore, standardized irisin assays and cfDNA measurement protocols would be needed to improve comparability across studies.

Although blood sampling was standardized in the morning after 24 h without structured training, we cannot exclude the contribution of hemoconcentration/hemodilution to part of the observed longitudinal changes. Future studies should incorporate markers of plasma volume to distinguish concentration/dilution effects from true changes in biomarker release or clearance.

Despite these limitations, our findings support the conceptual possibility of combining VO_2_max, irisin, and cfDNA to monitor training adaptations and recovery in elite youth ice hockey and can contribute to multi-parameter frameworks that help coaches to integrate physiological, molecular, and load-related information.

As a hypothesis-generating example, combined profiles of VO_2_max, cfDNA, and possibly irisin could correspond to distinct adaptation states (e.g., high VO_2_max, high irisin, and low cfDNA as a pattern consistent with good adaptation; high VO_2_max with high cfDNA as a profile compatible with residual stress; low VO_2_max with low cfDNA with under-stimulation).

## 5. Conclusions

In conclusion, seasonal training in elite U18 ice hockey players induced significant improvements in aerobic capacity and chronic irisin elevation, alongside reduced cfDNA and stable cortisol—reflecting positive physiological adaptation to a pre-season training regimen. These findings provide preliminary longitudinal evidence in adolescent team-sport athletes and highlight the value of integrating molecular biomarkers with traditional performance assessments in training monitoring.

## Data Availability

The original contributions presented in this study are included in the Article/Supplementary Material. Further inquiries can be directed to the corresponding author.

## Supplementary Materials

The following supporting information can be downloaded at: https://www.mdpi.com/article/10.3390/sports14020057/s1, Table S1: Shapiro–Wilk normality test for D and F group data; Table S2: Descriptive statistics and statistical significance of position-based differences between D and F groups; Table S3: Paired *t*-test parameters for longitudinal analysis; Figure S1: Schematic overview of study design, timelines and measurement domains; Figure S2: Distribution of VO_2_max response from pre-season to early-season (dVO_2_max) values.
