# Supplementary material for: Molecular and Physiological Adaptations to Seasonal Training in Elite U18 Ice Hockey Players
Source: Sports (Basel). 2026 Feb 4;14(2):57. doi: 10.3390/sports14020057 (PMC12944270; doi:10.3390/sports14020057)
Supplement: Supplementary file 1 [file sports-14-00057-s001.zip › sports-4067052-supplementary.pdf]

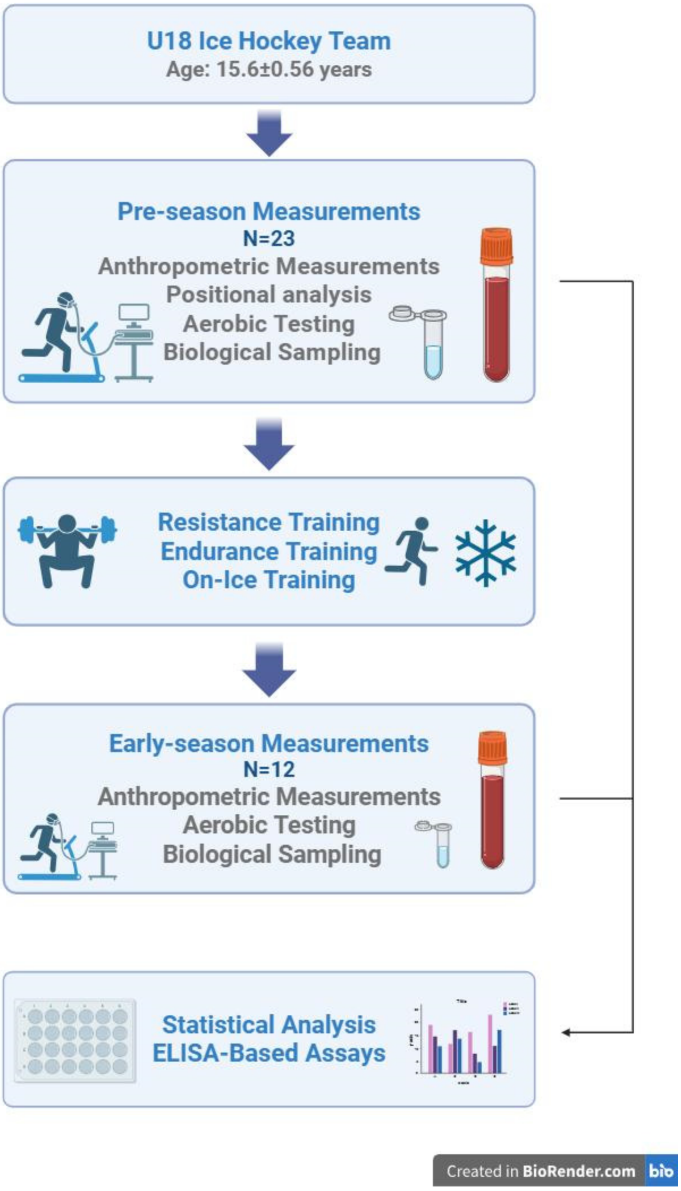

Figure S1. Schematic overview of study design, timelines and measurement domains.

Table. S1. Shapiro-Wilk normality test for D and F group data

Variable	Cortisol_D	Cortisol_F	VO_2 max _D	VO_2 max _F	Cell-Free DNA_D	Cell-Free DNA _F	Irisin_D	Irisin_F
**W-value**	0.88888	0.90709	0.90437	0.82731	0.82232	0.99189	0.93325	0.95956
**p-value**	0.3515	0.4503	0.4345	0.1328	0.1217	0.9859	0.6187	0.8049

Table. S2. Descriptive statistics and statistical significance of position-based differences between D and F groups

**Team n=23 (T)**

	Salivary Cortisol (ng/mL)	VO_2max (mL·kg⁻¹·min⁻¹)	Cell-Free DNA (ng/mL)	Irisin (ng/mL)
**Minimum**	1.119	43.1	1.381	586.4
**1st Quartile**	3.745	49.55	4.064	694.8
**Median**	6.304	52.5	6.516	755.4
**Mean**	7.598	52.25	10.913	860.6
**3rd Quartile**	10.366	54.9	10.787	922.5
**Maximum**	20.21	59	50.83	1763
**Standard Deviation**	4.917	3.941	12.743	269.745
**Defensemen n=5 (D)**				
	**Salivary Cortisol (ng/mL)**	**VO_2max (mL·kg^{-1}·min^{-1})**	**Cell-Free DNA (ng/mL)**	**Irisin (ng/mL)**
**Minimum**	1.715	48.7	3.615	586.4
**1st Quartile**	2.99	51.5	7.399	646.5
**Median**	5.323	51.9	8.114	693.1
**Mean**	5.668	52.84	18.888	683.2
**3rd Quartile**	9.041	53.1	24.485	739.8
**Maximum**	9.271	59	50.83	750.4
**Standard Deviation**	3.438	3.802	19.577	68.071
**Forwards n=14 (F)**				
	**Salivary Cortisol (ng/mL)**	**VO_2max (ml/min/kg)**	**Cell-Free DNA (ng/mL)**	**Irisin (ng/mL)**
**Minimum**	3.154	43.1	1.381	649.5
**1st Quartile**	4.792	50.1	3.965	732.7
**Median**	6.53	53.8	4.761	806.2
**Mean**	8.349	52.47	6.783	928.1
**3rd Quartile**	11.04	54.95	9.092	1052.2
**Maximum**	20.21	57.8	19.35	1763
**Standard Deviation**	4.989	4.226	4.846	310.334
**Goalies n=4 (G)**				
	**Salivary Cortisol (ng/mL)**	**VO_2max (mL·kg^{-1}·min^{-1})**	**Cell-Free DNA (ng/mL)**	**Irisin (ng/mL)**
**Minimum**	1.119	47.1	1.997	669.7
**1st Quartile**	2.012	48.52	4.4	721.7
**Median**	6.885	50.15	8.024	807.5
**Mean**	7.382	50.75	15.4	846.4
**3rd Quartile**	12.255	52.38	19.023	932.2
**Maximum**	14.64	55.6	43.555	1101
**Standard Dev.**	6.689	3.661	19.123	190.135
**F-D difference significance (one-way ANOVA)**				
**p-value**	0.3468	0.9444	0.1929	0.06118

**F-D effect size for comparisons**				
**Cohen's d**	0.73	-0.15	-1.51	1.2

Table S3. Paired t-test parameters for longitudinal analysis

Variable	t-value	p-value	95% CI	Mean sample pre-season	Mean sample early-season	Mean Difference (early-pre)	Hedges's g
**Cortisol**	-0.442	0.6668	-4.39 – 2.92	6.514	7.249	0.735	+0.12
**VO_2max**	-3.724	0.003359	-8.99 – -2.31	53.058	58.708	5.65	+1
**CF**	2.009	0.06974	-0.64 – 14.13	11.095	4.353	-6.742139	-0.54
**IR**	-3.109	0.009949	-180.75 – -30.90	718.729	824.553	105.824	+0.83

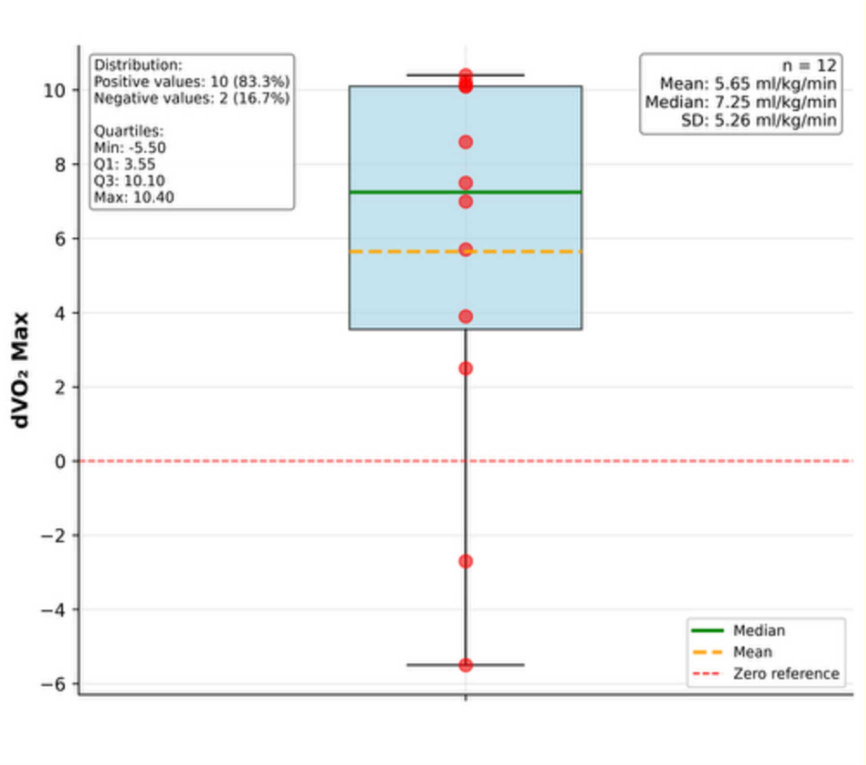

Figure S2. Distribution of VO_2max response from pre-season to early-season (dVO_2max) values. Box plot represents descriptive statistics and distribution data for dVO_2max. The red dashed line represents zero change.
